# Interpreting immune evasion: a novel assay for HLA loss detection

**DOI:** 10.3389/fimmu.2025.1603188

**Published:** 2025-07-01

**Authors:** Linnéa Pettersson, Sofia Westerling, Hamid Ramezanali, Francesco Vezzi, Rikard Eckerud, Dan Hauzenberger, Anders Hedrum, Jonas Mattsson, Michael Uhlin, Mehmet Uzunel

**Affiliations:** ^1^ Department of Medicine, Karolinska Institute, Stockholm, Sweden; ^2^ Devyser Aktie Bolag (AB), Stockholm, Sweden; ^3^ Viska AI AB, Stockholm, Sweden; ^4^ Division of Medical Oncology and Hematology, Princess Margret Cancer Center, University Health Network, Toronto, ON, Canada

**Keywords:** HSCT, NGS, HLA loss, immune evasion, haplo identical hematopoietic stem cell transplantation, MMUD-HSCT

## Abstract

This study presents the analytical performance of a new Next-Generation Sequencing (NGS) assay designed to detect Human Leukocyte Antigen (HLA) loss. Unlike existing methods, this assay offers increased sensitivity, broader applicability, and does not require prior knowledge of specific HLA mismatches, making it a more versatile tool for post-transplant monitoring. The main goal was to determine whether this assay can reliably identify HLA loss in post-transplant patients and provide clinically actionable information for relapse management. Furthermore, the clinical utility of the assay was assessed in patients undergoing Hematopoietic Stem Cell Transplantation (HSCT) with haploidentical or HLA-mismatched unrelated donors (MMUD). The study included both artificial and clinical samples, which were analyzed using the present assay to examine insertion-deletion (indel) markers located within and adjacent to the HLA region. The results demonstrated that the new assay exhibits excellent correlation with the One Lambda Devyser Chimerism assay in samples without HLA loss, achieving a detection limit of 0.25%. Furthermore, the study showed that the markers employed in the assay can effectively identify the occurrence and location of HLA loss. These findings could potentially influence clinical decision-making, when the donor source of retransplants or Donor Lymphocyte Infusions (DLI) need to be re-considered.

## 1 Introduction

Hematopoietic stem cell transplantation (HSCT) is the only curative treatment for many patients with malignant blood disorders. Typically, HSCT is performed using stem cells from an HLA-matched sibling donor or a matched unrelated donor (MUD) identified through international donor registries. However, the limitations of finding fully matched HLA donors, especially for ethnic minorities and multiethnic families, can be mitigated by using alternative donors such as related HLA-haploidentical (haplo) donors or HLA-mismatched unrelated donors.

The discovery on how to reduce Graft versus Host Disease (GvHD) in the setting of HSCT using HLA-haploidentical donors by altering the presence or function of T-cells, either by repleting or depleting these cells has been essential within the transplantation society (1, 2). This finding has led to a significant increase of transplantations with haploidentical or MMUD with similar outcomes as transplantations performed with siblings or matched unrelated donors (MUD) (3). However, as the number of haploidentical transplantations have increased (4), new mechanisms of immune escape and relapse have been discovered (5). One notable mechanism, known as HLA loss, was first described by Vago et al. in 2009 (6) and represents a significant form of immune evasion facilitating post-transplant relapses. In this process, recipient tumor cells become “invisible” to T-cells by losing their ability to express HLA antigens or complete HLA haplotypes on the cell surface. The lack of expression of recipient derived HLA antigens or haplotypes could result in promotion of immune escape of the patient’s malignancy from the surveillance by immune competent donor-derived T-cells (5). Initially observed in haploidentical transplantations, this mechanism was later also identified in HLA-mismatched unrelated donor transplantations (7). This discovery highlighted the broader relevance of HLA loss as a potential relapse causing mechanism across different types of hematopoietic stem cell transplantations. Several authors have suggested that up to 30% of all relapses in haplo-transplanted patients may be caused by genomic HLA loss (8). As awareness of HLA loss and its clinical implications have grown, there is now a recommendation for testing post-transplantation samples for both mixed chimerism and HLA loss (1, 9, 10).

The acute leukemia working party (ALWP) and the European Society for Blood and Marrow Transplantation (EBMT) have recommended testing for potential HLA loss at relapse before administering donor lymphocyte infusions (DLI) (9). However, clinicians have highlighted the lack of readily available methods to perform such tests (10, 11), which could improve the outcome of hematopoietic stem cell transplantation (HSCT).

In response to this shortcoming, we have developed a new, sensitive NGS assay that is user-friendly and can detect HLA loss when used in conjunction with a chimerism assay. This novel approach addresses the current limitations in post-transplant monitoring and has the potential to improve patient outcomes following HSCT.

## 2 Materials and methods

A total of 370 samples were tested for a total of 642 sample replicates to evaluate the performance of the HLA loss assay (**Table 1**, **Supplementary Figure 1**). 228 samples originated from 114 HSCT pairs of which 91 were haploidentical (haplo-matched) and 23 were mismatched unrelated donors (MMUD). Also 94 blood donor samples were included. The 114 matched HSCT pairs were obtained from Canada and Sweden. In addition, six cell-lines were included and mixed to create and simulate twelve artificial levels of chimerism (0.05-50% mixed chimerism) in three unique dilution series. Clinical post-transplant samples were available for 4 patients and comprised three cell-sorted sample fractions (CD3, CD33, and CD34) from one patient, a myeloid cell fraction for another patient along with two unsorted samples from two different patients with various malignancies.

**Table 1:** Samples used in evaluation of the HLA loss assay.

Sample type	Pairs	Samples	Replicates	Total
Pairs, Haplo	91	182	1	182
Pairs, MMUD	23	46	1	46
Blood donor		94	3	282
Dilution series 1		12	3	36
Dilution series 2		12	3	36
Dilution series 3		12	3	36
Cell-lines	3	6	3	18
Post transplant		6	1	6
**Total**		**370**		**642**

Artificial samples were used to assess the analytical performance including, Linearity, Limit of Detection (LoD), and Limit of Quantification (LoQ). The blood donor samples were used to assess the Limit of Blank (LoB) and the matched pairs were used to determine the informativeness of the indel markers. The post-transplant clinical samples were used as proof of principle for detection of HLA loss and to determine the clinical utility. The samples were collected according to the allocation rules applied at the Karolinska University Hospital as well as University Health Network (UHN), Toronto. Ethical approvals for analysis of clinical samples were obtained from the Ethical Review Board in Stockholm (2024-00185-02) and Research Ethics Board in Toronto (20-5134.4).

### 2.1 Artificial samples

To analyze Linearity, LoD, and LoQ, three unique artificial dilution series were made. Each dilution series was composed of two unique samples from Coriell Institute for Medical Research (Coriell), mixed in various ratios to simulate different levels of patient and donor chimerism. To simulate a haplo-transplantation pair the Coriell samples were selected based on containing only heterozygous informative markers. Each Coriell sample (NA02224, HG00232, HG01673, NA11992, HG00116, and HG00127) was diluted to 6 ng/µL using 0.1 x Tris-EDTA and quantified using Qubit HS following the manufacturer’s instructions (Invitrogen, Thermo Fisher Scientific). Each dilution series consisted of 12 samples for each set, with recipient chimerism ranging from 0.05% to 50%. Both the One Lambda Devyser Chimerism assay and the novel HLA loss assay were used to test these samples. The One Lambda Devyser Chimerism assay served as a reference method.

### 2.2 Clinical samples

Screening samples from haplotype matched and matched unrelated pairs were used to analyze the informativity of the assay and as proof of principle. Genomic DNA from whole blood samples from 91 haplotype matched pairs and 23 matched unrelated (10/18 to 19/20 match) were diluted to 3–6 ng/µL using 0.1 x Tris-EDTA and quantified using Qubit HS according to manufacturer instructions (Invitrogen, Thermo Fisher Scientific).

For one of the HSCT patients with relapse, the primary bone marrow sample was prepared into separate cell fractions (CD3+, CD33+, and CD34+) using immunomagnetic cell separation (IMS). DNA was automatically extracted (Hain Lifescience GMBH) using DiaSorin cell separation and DNA extraction reagents (LBH Advanced Bioservices) according to the manufacturer’s instructions. The chimeric status had previously been determined using an in-house short tandem repeat (STR) and quantitative PCR (qPCR) method (12, 13). The additional monitoring samples from three relapsing patients, originated from both bone marrow and whole blood, were diluted to 6 ng/µL and were tested with both One Lambda Devyser Chimerism and the HLA loss assay.

### 2.3 HLA loss analysis using NGS

**Figure 1:**
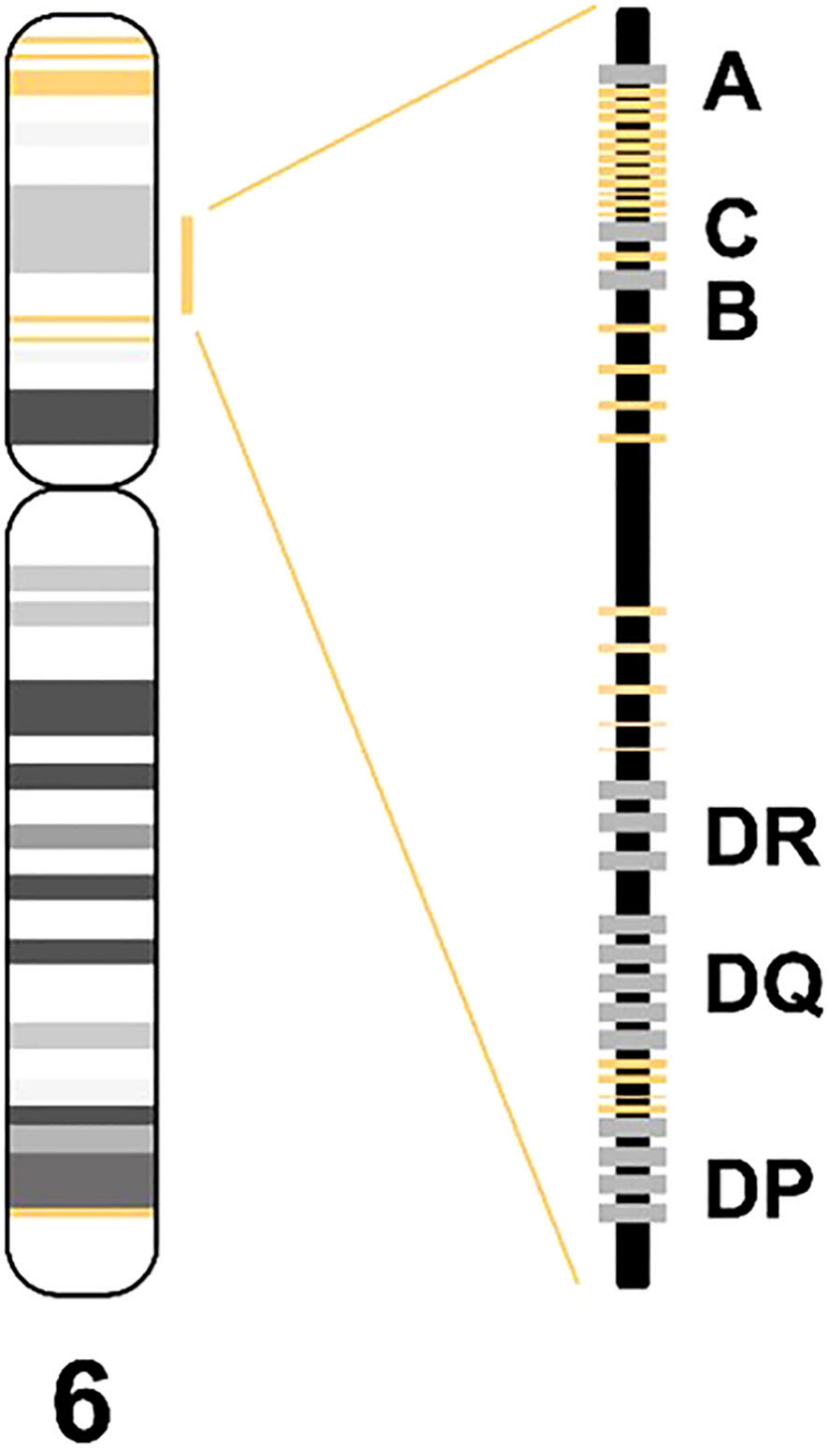
Schematic marker overview (yellow) in the HLA loss assay.

The HLA loss assay utilizes targeted sequencing to measure allele frequency of 26 indel markers within the HLA region and five indel markers flanking the HLA region on chromosome 6 (**Figure 1**). Twelve of the HLA markers are located between HLA-A and HLA-C, one is located between HLA-C and HLA-B, nine are located between HLA-B and HLA-DR, and four are located between HLA-DQ and HLA-DPA1. Three of the five flanking markers are located at 6p25.3, one at 6p12.2 and one at 6q27.

The indel markers included in the presented assay were designed and selected as described previously (14) (see **Supplementary Figure 2** for PCA plot of selected markers).

Each sample is amplified using a single multiplex PCR reaction containing marker-specific primer pairs to create a target amplicon library (PCR1). The target amplicon library is diluted and used as template in the second PCR (PCR2) where the adapters and unique indices are incorporated, enabling sample pooling prior to sequencing as well as sequencing the samples together with One Lambda Devyser Chimerism. The indexed libraries were pooled and purified using Devyser Library Clean according to manufactures instructions (Devyser AB, Sweden) and sequenced 2 x 75 cycles on Illumina MiSeq and MiniSeq Instruments, see **Supplementary Figure 3** for schematic system workflow.

The resulting indel marker PCR products (amplicons) have an average size of 67 bp (including target specific primer) and a maximum size of (with insertion and target specific primers) of 79 bp, enabling almost full coverage using 2 x 75 cycles of sequencing.

### 2.4 Chimerism and HLA loss calculations

**Figure 2:**
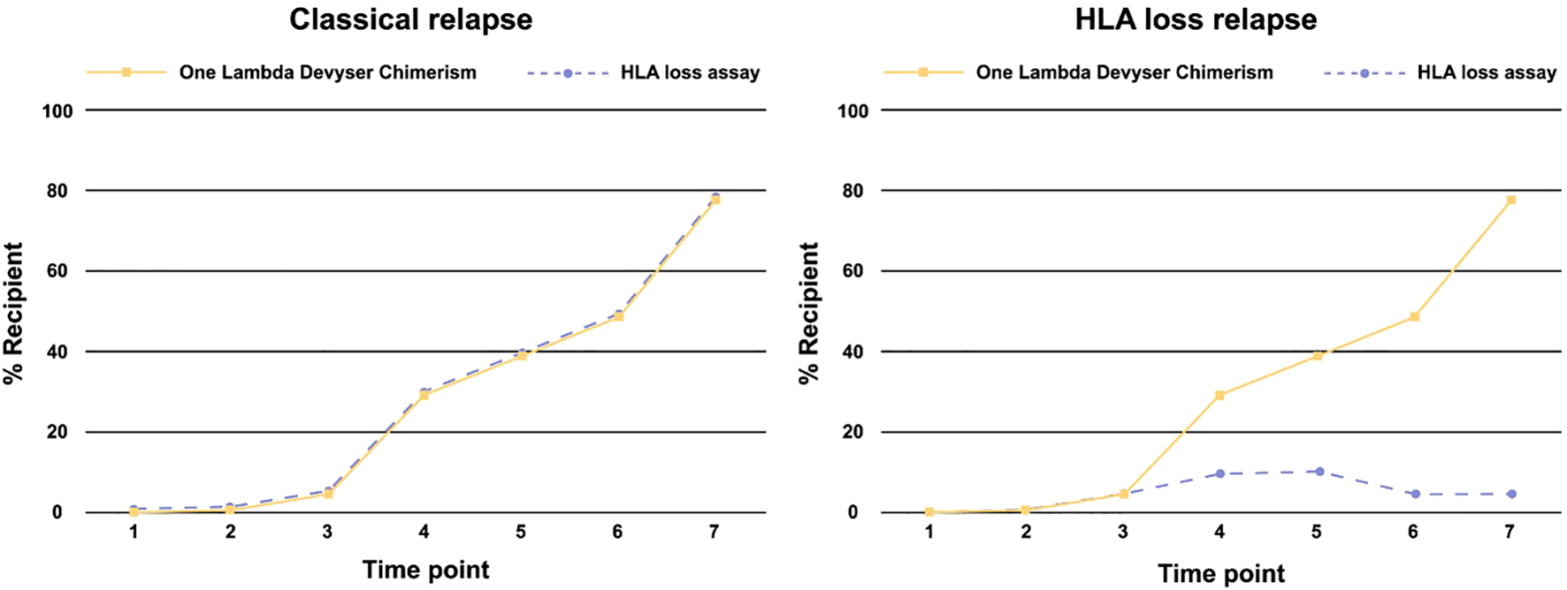
Schematic of a classical relapse and an HLA loss relapse in relation to chimerism.

To identify a genomic HLA loss, the chimerism detected in the HLA region is compared to the chimerism observed for other chromosomes of a post-transplant sample. This assessment typically involves using methods like One Lambda Devyser Chimerism to establish the baseline chimerism across non-HLA chromosomes. If the chimerism levels detected by the HLA loss assay conforms with those found in other chromosomes, it suggests that no HLA loss has occurred (*i.e.*, a classical relapse). If the HLA loss assay instead deviates and exhibits significantly lower chimerism levels compared to other chromosome regions, this indicates potential loss of HLA. (as illustrated in **Figure 2**).

The software and variant allele frequency (VAF) calculations used in this analysis are based on methods previously detailed in Pettersson et al., 2021 (14). Also, to compensate for a potential bias between the long and short allele amplification, a correction value is calculated based on the VAF in heterozygous markers. The correction value was calculated from blood donors containing one genotype and was then applied on the VAF in the monitoring sample. A key distinction between the software employed for One Lambda Devyser Chimerism and for the HLA loss assay is the presentation of results. Instead of providing an average chimerism value, the HLA loss software reports chimerism values individually for each informative marker. This approach is particularly relevant given the recent findings, as reviewed by Arnold in 2023 (15), which highlight that different segments of the HLA region may be subject to loss. The markers in the HLA loss assay are designed to capture these potential localized losses within the HLA region. By presenting chimerism data for each marker separately, the software allows for a more nuanced understanding of potential HLA loss patterns, reflecting the complex nature of genetic changes in this region.

### 2.5 Statistical analysis

All statistical analyses were conducted using R, an open-source programming language for statistical computing [R Core Team, 2023 (https://www.r-project.org)]. Data visualization was carried out using RStudio version 2024.09.0 build 375. Pearson’s least squares method was employed for calculating correlation and regression.

### 2.6 Analytical performance

The analytical performance of the HLA loss assay was evaluated for the following parameters: LoB, LoD, LoQ, trueness, and linearity.

LoB was established using single-genotype samples, typically pre-transplantation samples. The analysis involved 282 measurements, corresponding to 78652 theoretical blank markers.

LoD, defined as the lowest reliably distinguishable chimerism percentage from LoB, was calculated following CLSI EP17-A2 guidelines. Using the dilution series, each low level sample was tested in triplicate with one batch of the HLA assay, totaling 63 measurements. This calculation focused on heterozygous markers, being particularly relevant in the context of haplo-donor transplantations. LoQ was determined as the lowest measured mixed chimerism at or above LoD with a coefficient of variation (CV) < 20%. The dilution series with mixed chimerism at or over LoD was used, with three replicates totaling 45 measurements.

Trueness and linearity were assessed by comparing results to the One Lambda Devyser Chimerism, specifically its measurements of chimerism for chromosomes other than chromosome 6 as reference method. Three dilution series ranging from 0.05% to 50% were tested in replicates using both assays, resulting in 108 measurements for each assay. For more information see **Supplementary**.

## 3 Results

### 3.1 Analytical performance

The analytical performance of the HLA loss NGS assay was evaluated, focusing on key parameters: LoB, LoD, and LoQ. LoB was defined as the 95th percentile of the average measured background in blank samples and was determined to be 0.2%, with an average of 0.05%, when considering all informative markers.

To assess the assay’s sensitivity, the LoD and LoQ were evaluated using artificially mixed samples with varying levels of MC. The analysis method is detailed in the materials and methods section. For all informative heterozygous markers, both the LoD and LoQ were determined to be 0.25% and 0.3% respectively.

These results indicate that the assay is capable of detecting and quantifying very low levels of MC compared to, for example using STR and capillary electrophoresis, that exhibits low sensitivity but high precision in cases with high amounts of mixed chimerism (14).

**Figure 3:**
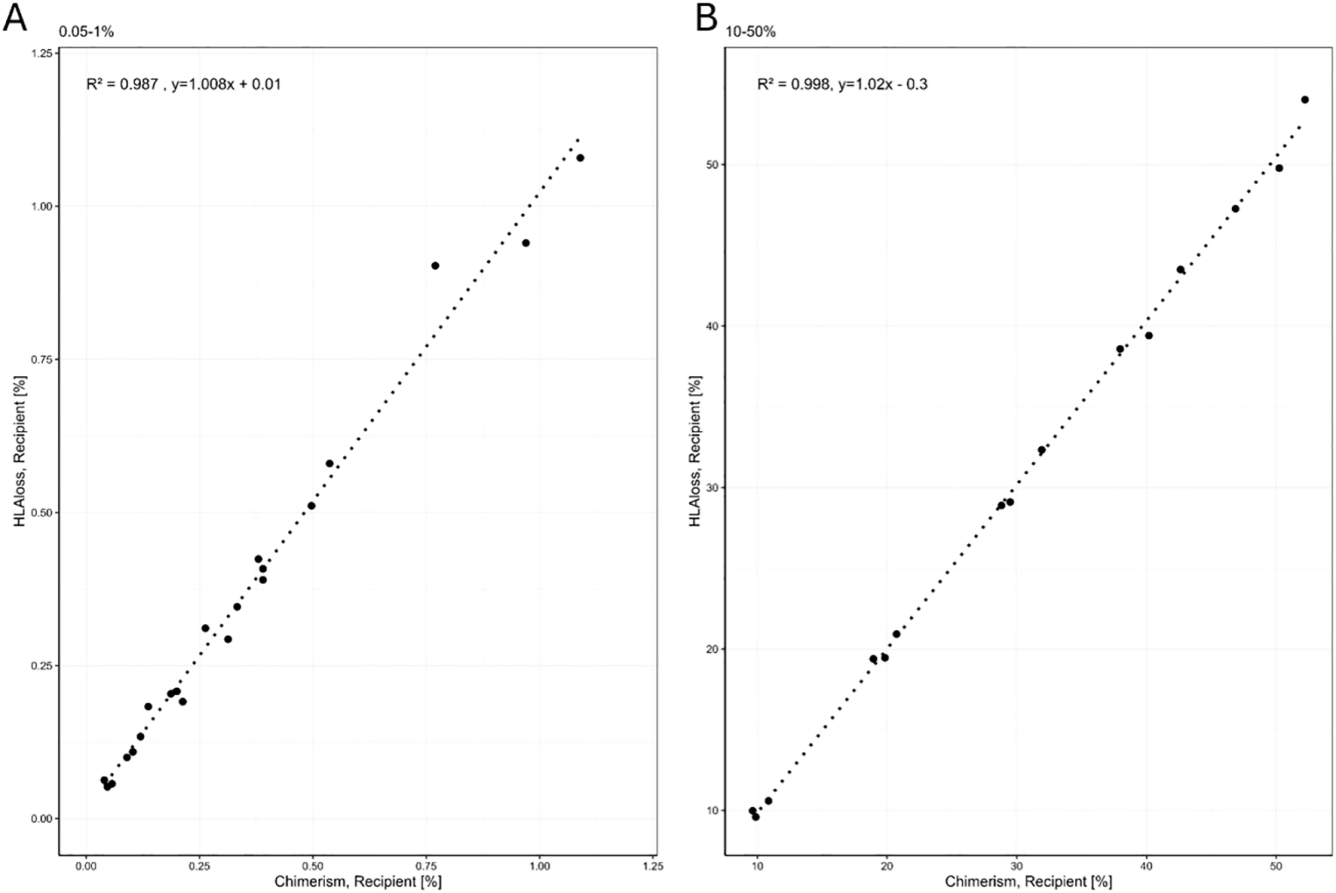
Linear regression using Pearson correlation between %MC measured with One Lambda Devyser Chimerism and the HLA loss assay. **(A)** regression of MC between 0.05-1%. **(B)** regression between 10-50% MC.

The assay’s range of quantification, linearity, and trueness was evaluated using a series of dilution points. Each point in the dilution series was tested in triplicate, and the average percentage was calculated. These results were then compared with the average chimerism detected by One Lambda Devyser Chimerism, as illustrated in **Figures 3A, B**.

The data from all three dilution series were combined, and the average of all replicates was calculated. For analysis purposes, the results were divided into two ranges: high (10-50% MC) and low (0.05-1% MC).

To assess linearity, a linear regression analysis was performed on each individual series. All series demonstrated R² values exceeding 0.99, indicating a high degree of linearity across the tested range. The R² values were 0.998 for the upper range and 0.987 for the lower range, demonstrating strong correlation and linearity across both ranges. These data demonstrate that the assay performs linearly and accurately with minimal systematic bias across both mixed chimerism ranges.

### 3.2 Clinical utility

#### 3.2.1 Informativity

**Figure 4:**
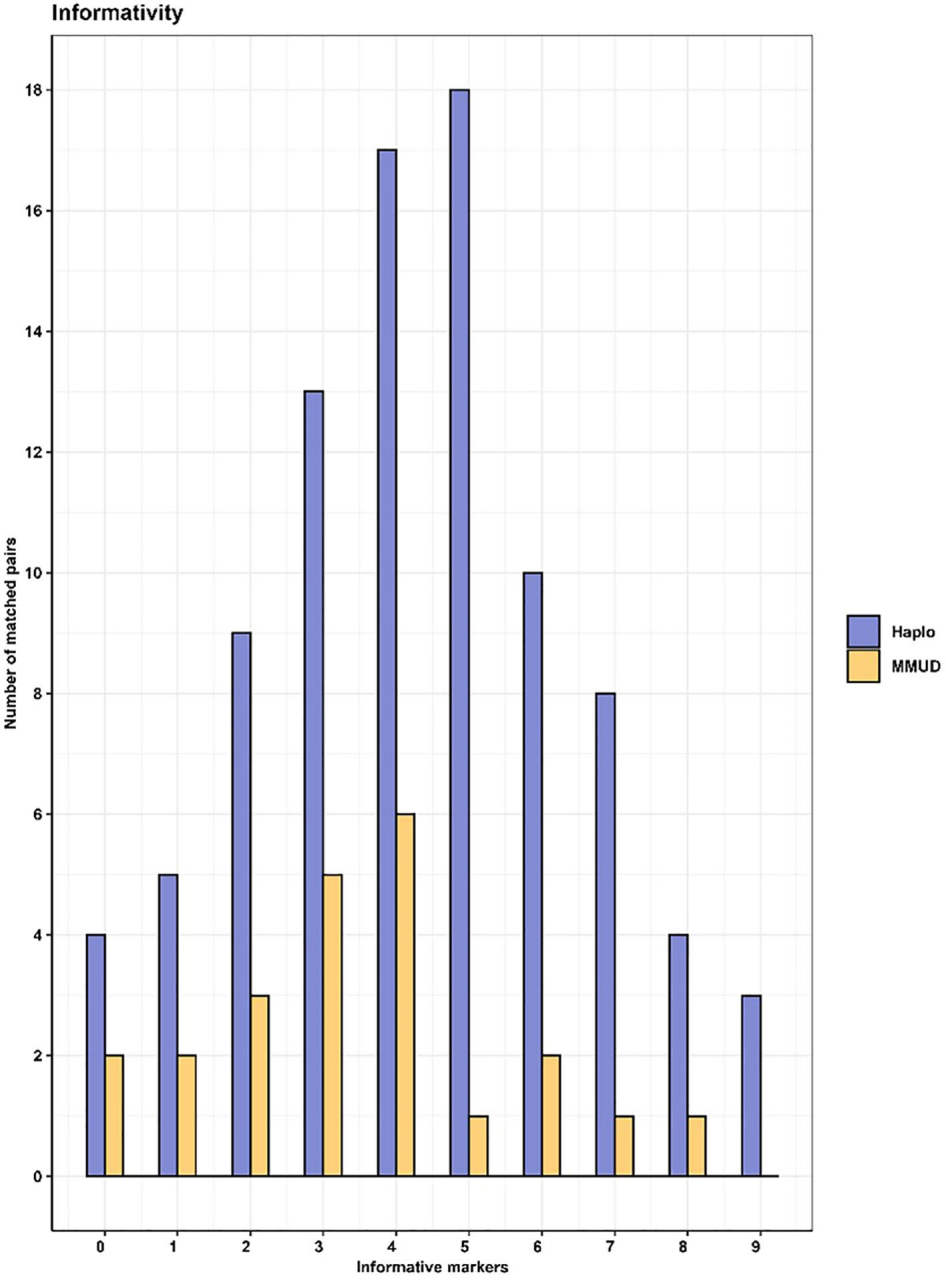
Number of informative markers in haplo (purple) -and MMUD pairs (yellow).

In the haplo-matched pairs, only heterozygous informative markers were detected, as expected. However, we observed no significant difference in the number of informative markers when comparing haplo-matched and MMUD pairs. In six (5.2%) of the 114 matched pairs analyzed, no informative markers relating to the patient could be identified. However, of the six matched pairs, informative markers relating to the donor were identified in four of them, while the remaining two pairs shared identical genotypes for all markers in the assay. In a few cases, where the patient fraction is high, the markers identified for the donor can be used to detect HLA loss resulting in only two pairs (1.8%) without any informative markers. For the pairs with identical genotypes, a traditional HLA sequencing might be the solution or examine the loss of heterozygosity that is shown later. On average, we identified four informative markers per pair, with a range of 0 to 9 markers. The frequency distribution of informative markers is illustrated in **Figure 4**.

#### 3.2.2 Patient cases and clinical utility

To demonstrate the assay’s clinical utility, we selected four samples from patients experiencing clinical relapses. These cases were chosen to showcase the diverse advantages of the method. The samples represent patients with four distinct diagnoses and were obtained from various sources: cell-separated bone marrow fractions, whole blood, and non-cell separated bone marrow. Additionally, the donors prior to HSCT included both mismatched unrelated donors and haplo-matched donors.

##### 3.2.2.1 Patient 1

A 56 year old male patient diagnosed with AML, transplanted with a HLA-haploidentical donor, relapsed 1334 days after transplantation date. The patient had a bone marrow sample taken at the time of relapse, which exhibited a mixed chimerism in the separate cell fractions (CD3 0.02%, CD33 4%, and CD34 85%).

**Figure 5:**
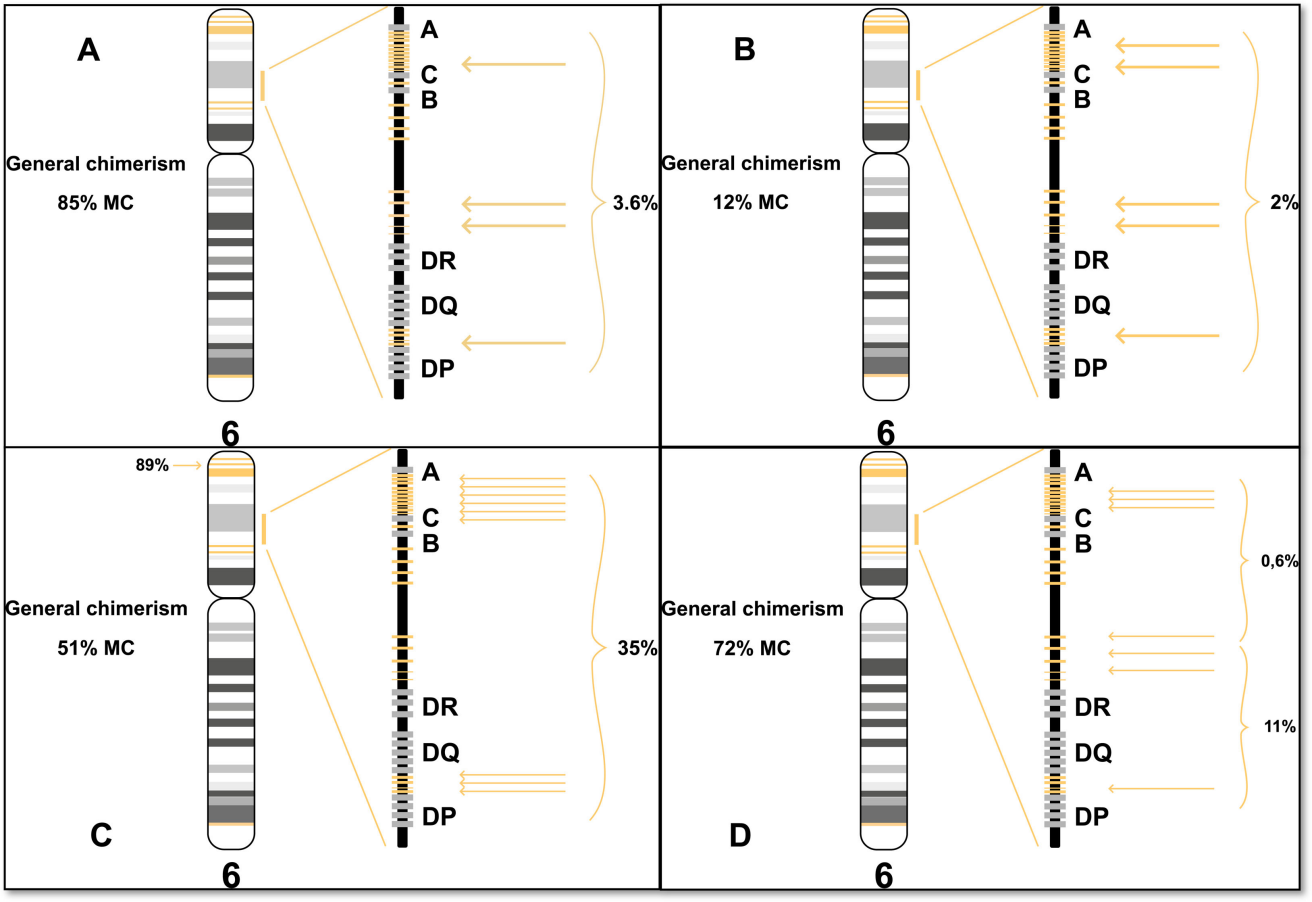
HLA region recipient chimerism and non-chromosome 6 chimerism (left of chromosome) in patient samples. **(A)** Patient 1: Four informative markers reveal varying chimerism percentages across different cell fractions, here we show the CD34 fraction. **(B)** Patient 2: Five informative markers consistently show 2% chimerism in the HLA region, while other chromosomes exhibit 12% MC. **(C)** Patient 3: Ten informative markers indicate 35% chimerism in the HLA region, except for a marker on the p-arm end showing 89% chimerism. Other chromosomes display 51% MC. **(D)** Patient 4: Seven informative markers split between 0.6% chimerism and 11% MC in the HLA region. Other chromosomes show 72% recipient chimerism.

Analysis of these samples, using the Devyser HLA loss assay, identified four recipient specific informative markers, five markers heterozygous for both donor and recipient, and four informative markers specific for the donor. Two of the recipient specific informative markers were located between HLA-A and HLA-C, while the other two were located between HLA-B and HLA-DR-A (**Figure 5A**). Heterozygous markers originating from both recipient and donor were identified throughout the analyzed region (HLA-A to HLA-DPA1).

**Figure 6:**
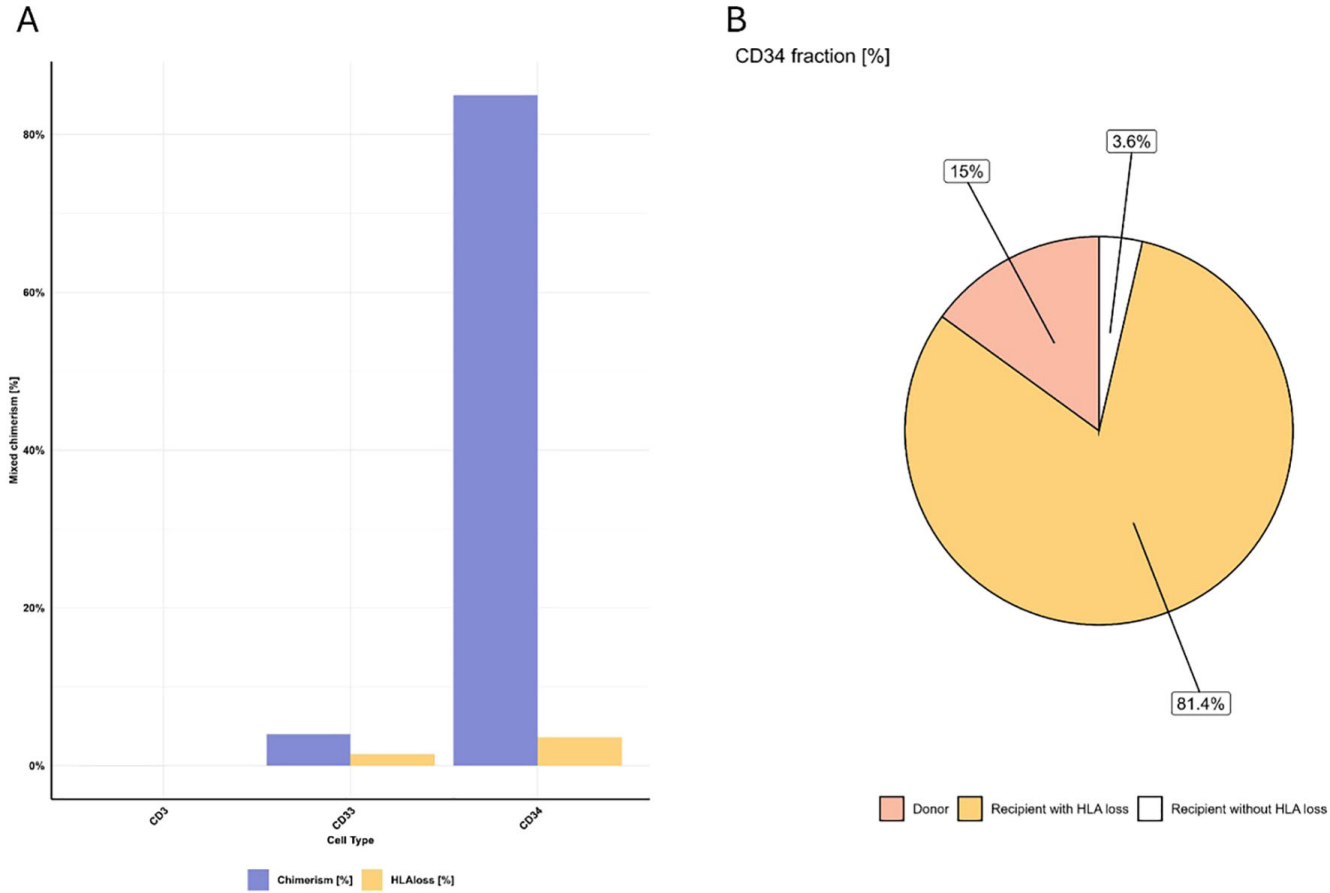
Results from patient 1. **(A)** Recipient levels using chimerism (detected with STR/qPCR, blue) and HLA loss assay (yellow) in three cell fractions. **(B)** Origin distribution within the CD34 fraction. 15% originated from donor, 3.6% originates from the recipient with an intact HLA region and 81.4% originates from recipient with a loss of heterozygosity.

The HLA loss assay could not detect any mixed chimerism in the T-cell fraction, but detected 1.5% recipient in the myeloid fraction, and 3.6% recipient in the hematopoietic stem cell fraction (**Figure 6A**).

The detection of low-level MC in the CD34 fraction indicated that not all recipient cells had lost the complete HLA haplotype. Of the cells in the CD34 fraction, 15% originated from the donor, 81.4% were recipient cells that had lost the HLA region, and 3.6% were recipient cells with an intact HLA region (**Figure 6B**). The combination of information of the recipient markers and the heterozygous markers indicate that one whole HLA haplotype was lost.

##### 3.2.2.2 Patient 2

A female patient diagnosed with acute lymphoblastic leukemia (ALL) underwent chimerism analysis of a bone marrow sample taken 97 days post HSCT, at the same time as the confirmed relapse. One Lambda Devyser Chimerism assay revealed a 12% mixed chimerism in the sample. However, when the same sample was subsequently analyzed with the Devyser HLA loss assay, only 2% chimerism was detected. A total of five informative markers distributed across the HLA region were identified using the Devyser HLA loss. Two of the informative markers were located between HLA-A and HLA-C and two were located between HLA-B and HLA-DR. The fifth informative marker was located between HLA-DQ and HLA-DP (**Figure 5B**). Notably, all five of these informative markers indicated HLA loss in the sample. This consistent pattern across all markers suggests a significant loss of heterozygosity in the HLA region of the recipient’s cells. The discrepancy between the chimerism percentages detected by the two different assays, coupled with the evidence of HLA loss, requires further investigation and may have important implications for the patient’s treatment and prognosis.

##### 3.2.2.3 Patient 3

A male patient who had previously been diagnosed with myelodysplastic syndrome (MDS), underwent full conditioning as was transplanted with a HLA-mismatched unrelated donor. The patient had a bone marrow verified relapse 97 days post HSCT and analysis of the myeloid fraction from a blood sample (taken 26 days prior to relapse) was performed using the One Lambda Devyser Chimerism assay. The analysis exhibited a MC of 51% across most markers. However, a notable exception in the form of an 89% mixed chimerism that was observed in one marker located at the end of the p-arm of chromosome 6 (6p25.3).

Further investigation, using the Devyser HLA loss assay, demonstrated that the sample had a 35% mixed chimerism in the HLA region. This assay identified ten informative markers, where seven were located between HLA-A and HLA-C, and three were located between HLA-DQ and HLA-DP (**Figure 5C**).

These data suggest that a complete HLA-haplotype was lost in a fraction of the recipient cells present in the sample. Additionally, the high chimerism percentage observed at the end of the p-arm suggest a possible duplication in this region.

##### 3.2.2.4 Patient 4

A bone marrow sample was obtained from a female patient diagnosed with myelofibrosis at the date of relapse, which was 236 days post HSCT with a haplo-donor. Initial analysis exhibits a mixed chimerism of 72%, indicating a significant presence of recipient genotype. The HLA loss assay identified seven informative markers distributed across the HLA region. Specifically, three markers were located between HLA-A and HLA-C, three between HLA-B and HLA-DR, and one between HLA-DQ and HLA-DPA1. All three markers between HLA-A and HLA-C, as well as one marker between HLA-B and HLA-DR, showed a chimerism of 0.6%. The remaining markers demonstrated an elevated chimerism of 11% (**Figure 5D**). This variation in chimerism percentages across different markers suggests the presence of several subpopulations of recipient cells in the sample, each with distinct breakpoints in the HLA region. Based on these results, it appears that approximately 85% of the recipient cells (which constitute 72% of the total sample) may have lost the entire HLA haplotype. Another 14% of the recipient cells seem to have lost half of one HLA haplotype (more specifically, the HLA class I genes), while 0.8% of the recipient cells maintain an intact HLA region. Additional analysis of markers in both the p-arm and q-arm of chromosome 6 indicates that the genetic alterations are confined to the HLA region, with no evidence of loss or gain in the surrounding chromosomal areas. See **Figure 5D**.

#### 3.2.3 Non-informative markers

**Figure 7:**
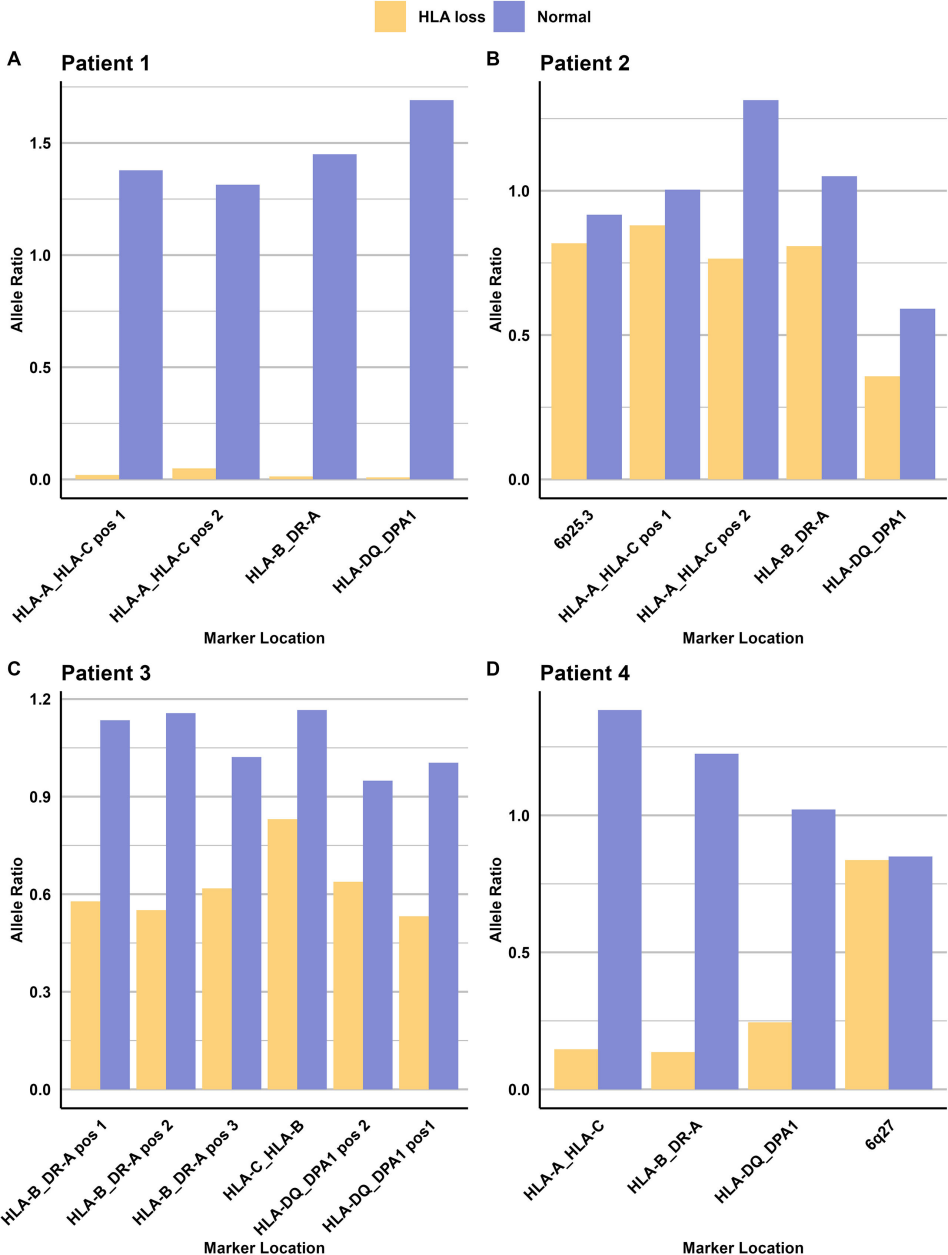
Allele ratios of heterozygous ‘non-informative’ markers in HLA loss analysis. Normal allele ratio is based on heterozygous markers from blood donors only containing one genome. **(A)** Patient 1 (CD34 fraction): Complete loss of heterozygosity in all four heterozygous markers. **(B)** Patient 2: Decreased allele ratio across all five heterozygous markers. **(C)** Patient 3: Reduced allele ratio in all six heterozygous markers. **(D)** Patient 4: Decreased allele ratio in all markers except one located on the q-arm, suggesting selective loss of the HLA region.

In traditional mixed chimerism analysis, markers may be classified as non-informative when both donor and recipient share the same genotype (e.g., both homozygous for an insertion or deletion, or both heterozygous). However, heterozygous markers showed to be valuable in loss of heterozygosity (LOH). Typically, heterozygous markers exhibit an allele ratio between 0.5 and 2, depending on the specific marker. In patients demonstrating genomic HLA loss, this ratio becomes skewed. For instance, in Patient 1’s CD34 fraction, the allele ratio ranges from 0.01 to 0.05. Similar patterns are observed in other patients (refer to **Figure 7**). This information can be used together with the informative markers to get a fuller picture of the biological events.

## 4 Discussion

The primary objective of this study was to evaluate a diagnostic assay capable of detecting LOH within the HLA-region in samples post HSCT. The present assay demonstrates eligible clinical utility and the precision and sensitivity necessary for accurate quantification and detection of HLA loss in a clinical setting. One of the key advantages of this assay is its rapid turnaround time, as it can produce results within 2 days, from DNA extraction to final analysis and is not patient/donor match dependent.

Other assays that are used for detecting HLA loss are based on typing HLA and using different markers for each patient and donor pair, depending on the mismatches. The markers in this assay are located between the different HLA genes that are infrequently included in standard HLA typing. We have also demonstrated that markers considered ‘non-informative’ in previous approaches can provide valuable information, presented in **Figure 7**. This approach would also be beneficial in patient and donor pairs when no ‘classically informative markers’ can be identified. It’s important to note that this approach can only be used when mixed chimerism levels are high (approximately >20% Recipient) and the loss occurs in a large proportion of the recipient cells, The Limit of Detection for this needs to be evaluated further In our cohort, this assay was applicable in 95% of patient pairs, which may reflects its utility in a clinical setting. For the remaining cases where this assay is not suitable, traditional HLA sequencing and the use of mismatched alleles as markers may be necessary. By using markers across the different HLA classes, it is possible to analyze whether the whole HLA complex is lost or only parts of it, as illustrated in **Figure 5**. This partial loss may be a result from selective immune pressure, where donor-derived T cells specifically target mismatched HLA alleles, leading leukemic clones to lose only those targeted regions while retaining others to evade immune detection. The ability to identify the type of HLA loss in a patient has significant clinical implications. When HLA loss is detected, clinicians can promptly begin searching for a new donor—such as another haplo-identical donor, as suggested by Luca Vago and Fabio Ciceri 2017 (16), or one matched for the specific HLA class that has been lost, depending on the results. Another intervention, suggested by Arnold 2022, is NK cell therapy that can target cells missing HLA class I (15). As only a single case of partial HLA loss was observed in our cohort, we are unable to draw any conclusions regarding the relationship between HLA mismatching and HLA loss in this context. However, this observation highlights an interesting area for future research, particularly now that accessible and reliable tools are available to facilitate such studies.

Moreover, the information can guide treatment decisions, particularly regarding DLIs. In cases where HLA loss is confirmed, DLI should be reconsidered, as it potentially may lack effect on the reemerging malignancy and subsequently on patient survival.

The consensus recommendation, as outlined by Li et al. in 2018, suggested investigating HLA loss at relapse in patients who have undergone haploidentical transplantation. However, implementing this recommendation has been challenging for most transplant centers due to the lack of a standardized methodology (17). These findings have highlighted the growing need for an easy-to-use and accurate assay to detect HLA loss. NGS assays have previously demonstrated high sensitivity in detecting low percentages of MC as well as accuracy in measuring high percentages of MC, as reported previously (14) and is usually accessible within a HLA lab.

As a result of the accessibility, many transplant centers have been transitioning from traditional methods such as STR analysis and qPCR to NGS-based approaches for chimerism assessment and can in extension perform HLA loss detection. This advancement has the potential to enhance the identification and management of post-transplant complications, particularly relapses associated with HLA loss.

This novel NGS assay, coupled with its accompanying software, offers an integrated solution for HLA loss detection. The software automatically selects the informative genetic markers for analysis. Furthermore, the assay has the capability to identify allelic imbalances in heterozygous markers, including those previously considered ‘non-informative’, which can indicate HLA loss. This approach enhances the sensitivity and specificity of HLA loss detection in post-transplant relapse scenarios. The assay is designed for use in clinical laboratories and can be seamlessly integrated with chimerism analysis using established methods. This combination makes it particularly valuable for the clinical management of transplant patients. Rapid turnaround times for results enable clinicians to make informed decisions within a clinically relevant timeframe, potentially impacting treatment strategies for patients with post-transplant relapse.

Our study demonstrates the versatility of the assay across various sample types, including whole blood, bone marrow, and cell-separated samples. This flexibility is particularly valuable given the well-documented discrepancies between chimerism results in blood and bone marrow samples reported by numerous transplant centers (13, 18–20) and the different sample sources that is regularly used within a chimerism lab (10). Furthermore, our findings expand the spectrum of hematological malignancies associated with HLA loss beyond the typically implicated AML (21, 22) and MDS (23–25) with the two patients that had initial diagnosis of MF and ALL. We observed HLA loss in patients initially diagnosed with myelofibrosis and acute lymphoblastic leukemia, suggesting a broader applicability of HLA loss investigation. Even though the limitation of our restricted sample cohort, the proof-of principle samples reveals that the assay is equally effective for patients who underwent transplantation from mismatched unrelated donors or haploidentical donors (**Figure 4**), further emphasizing its wide-ranging utility in post-transplant monitoring.

Several studies, together with this one, have demonstrated that it is not always the whole HLA complex that is lost but rather parts of it. In 2004, Rimsza et al. found that varying expression of MHC class II genes had a big effect on overall survival in diffuse large B-cell lymphoma (DLBCL) (26), in extension one would assume that the loss of this region also would have a big effect, but this needs to be investigated further together with the clinical background and outcome.

In summary, we demonstrate the assay’s clinical utility and highlight its short turnaround time. By providing a sensitive tool for rapid and accurate HLA loss detection without the need for HLA mismatch information, this assay represents a valuable addition to the arsenal of post-transplant monitoring techniques, potentially improving patient outcomes through earlier detection of complications.

## Data Availability

The raw data supporting the conclusions of this article will be made available by the authors, without undue reservation.
