# Supplementary material for: Interpreting immune evasion: a novel assay for HLA loss detection
Source: Front Immunol. 2025 Jul 1;16:1603188. doi: 10.3389/fimmu.2025.1603188 (PMC12259423; doi:10.3389/fimmu.2025.1603188)
Supplement: Supplementary file 1 [file DataSheet1.docx]

**Supplemental**

**Method:**

**2.4 Sequencing data analysis and quality control**

All sequencing reads that passed quality assessment were mapped to the target amplicons using a k-mer-based seed-and-extend approach (k=5). Following this, each read (read1 and read2) was aligned to both the reference and alternate sequences using a modified Smith-Waterman algorithm. A read pair was used if both reads matched either the reference or alternate sequence—allowing up to three mismatches and a single base indel and if their alignments were concordant. Such pairs were then assigned to the appropriate amplicon and haplotype (reference or alternate) and included in the chimerism calculation. Read pairs that did not meet these criteria (e.g., discordant alignments between read1 and read2, or alignment to an unrecognized haplotype) were excluded from analysis and classified as noise. If a sample lacked any informative markers meeting the required minimum number of paired reads, it was deemed a failed sample.

To ensure the reliability of the results, strict quality control thresholds were set for inclusion in the analysis. Specifically, samples were excluded if they did not contain informative markers with sufficient paired read depth, defined as at least 100 paired reads for pre-transplant samples and 5,000 paired reads for post-transplant samples. The 5,000 paired-end read threshold for post-transplant samples was chosen to achieve a detection sensitivity for chimerism down to 0.25%, corresponding to a minimum of 13 minor allele reads and a coverage depth of at least 10X.

**Analytical performance**

***2.7.1 Limit of blank (LoB)***

The Limit of Blank (LoB) was established using samples containing a single genotype, typically represented by pre-transplantation samples. LoB was defined as the average measured background of a secondary genotype detected in the informative markers across all theoretical pair combinations in these blank samples.

To determine the LoB, a comprehensive analysis was conducted using 282 measurements, which corresponded to a total of 78652 theoretical blank markers.

***2.7.2 Limit of detection (LoD)***

The Limit of Detection (LoD) was defined as the lowest chimerism percentage that could be reliably distinguished from the LoB. To determine the LoD, we utilized the previously established LoB and tested replicates of chimerism samples known to contain low amounts of DNA from a second genotype.

The LoD calculation followed the CLSI EP17-A2 guidelines and was expressed as:

$$LoD=LoB+c_{p}SD_{L}$$

Where SD_L_ represents the standard deviation of all replicates pooled across unique low-level samples, and cₚ is a multiplier reflecting the degrees of freedom of SD_L_.

For the LoD study, we used a sample set comprising the following recipient chimerism levels: 0.05%, 0.1%, 0.2%, 0.3%, 0.4%, 0.5%, and 1.0%. Each sample was tested in triplicate using one batch of the HLA assay, resulting in a total of 63 measurements. This calculation was performed for heterozygous markers, being particularly relevant in the context of haplo-donor transplantations.

***2.7.3 Limit of quantification (LoQ)***

The Limit of Quantification (LoQ) was established as the lowest measured mixed chimerism (%MC) that meets a predefined accuracy goal. Specifically, the LoQ was defined as the lowest level of %MC measured at or above the LoD with a coefficient of variation (CV) of less than 20%.

To determine the LoQ, we used a comprehensive sample set comprising the following chimerism levels: 0.2%, 0.3%, 0.4%, 0.5%, and 1%. Each sample was tested in triplicate using one batch of One Lambda Devyser Chimerism and one batch of the HLA loss assay, resulting in a total of 45 measurements.

***2.7.5 Trueness***

The accuracy of an assay is primarily determined by its trueness, which is the degree of agreement between the measured result and an accepted reference value. In the case of Devyser HLA loss, a semi-quantitative assay designed to measure chimerism in post-transplantation samples, trueness is assessed by comparing its results to those obtained from a reference method. For this study, the reference method chosen was One Lambda Devyser Chimerism, specifically its measurements of chimerism for chromosomes other than chromosome 6. The trueness of the Devyser HLA loss assay is evaluated based on how closely its results agree with this reference for the same samples. To conduct the trueness study, a set of samples with varying levels of MC were used. The sample set included the following chimerism percentages: 0.05%, 0.1%, 0.2%, 0.3%, 0.4%, 0.5%, 1.0%, 10%, 20%, 30%, 40%, and 50%. Each sample was tested in triplicate using both one batch of One Lambda Devyser Chimerism and one batch of Devyser HLA loss, resulting in a total of 108 measurements with each assay.

***2.7.7 Linearity***

For the linearity study, a comprehensive range of mixed chimerism (MC) samples was employed. The sample set included the following chimerism percentages: 0.05%, 0.1%, 0.2%, 0.3%, 0.4%, 0.5%, 1.0%, 10%, 20%, 30%, 40%, and 50%. To ensure reliability and account for potential variability, each sample was tested in triplicate. All tests were conducted using a single batch of the Devyser HLA loss assay and a single batch of One Lambda Devyser Chimerism that was used as reference value. This. approach resulted in a total of 108 measurements for each assay (12 chimerism levels × 3 replicates × 3 dilution series).


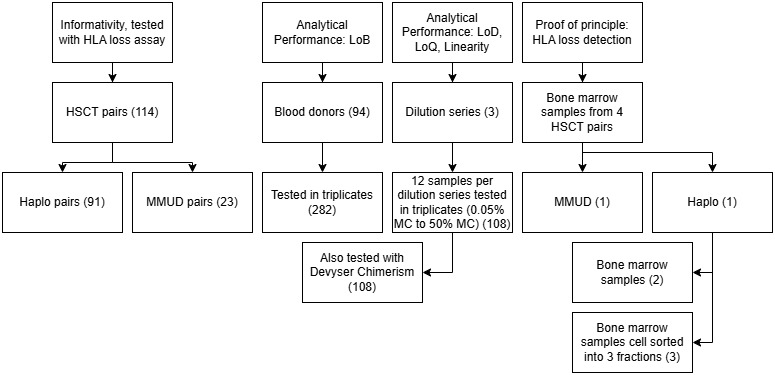
Supplemental figure 1. Flowchart of samples used in each experiment


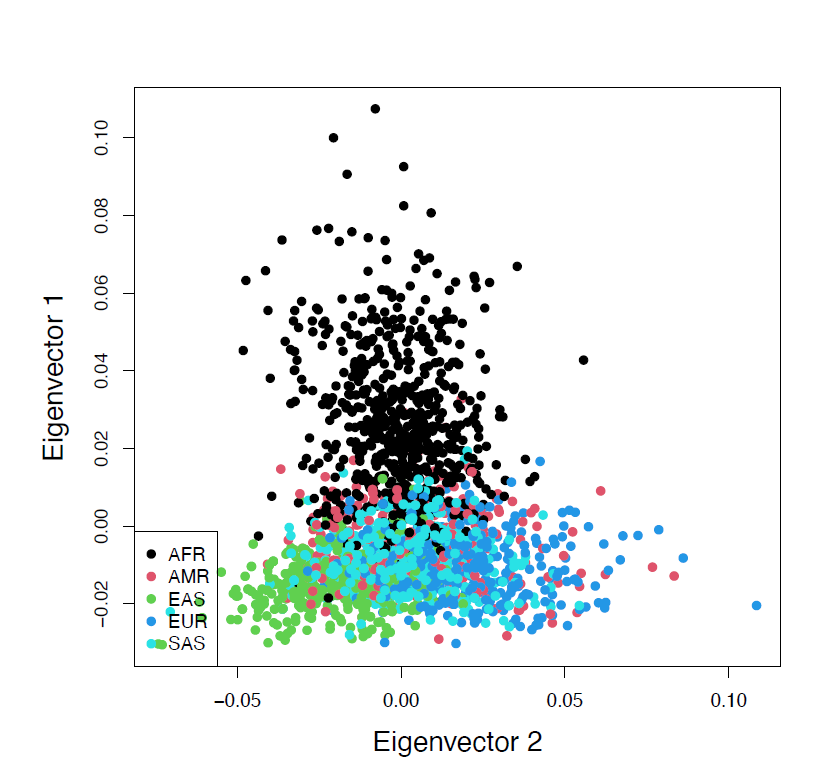

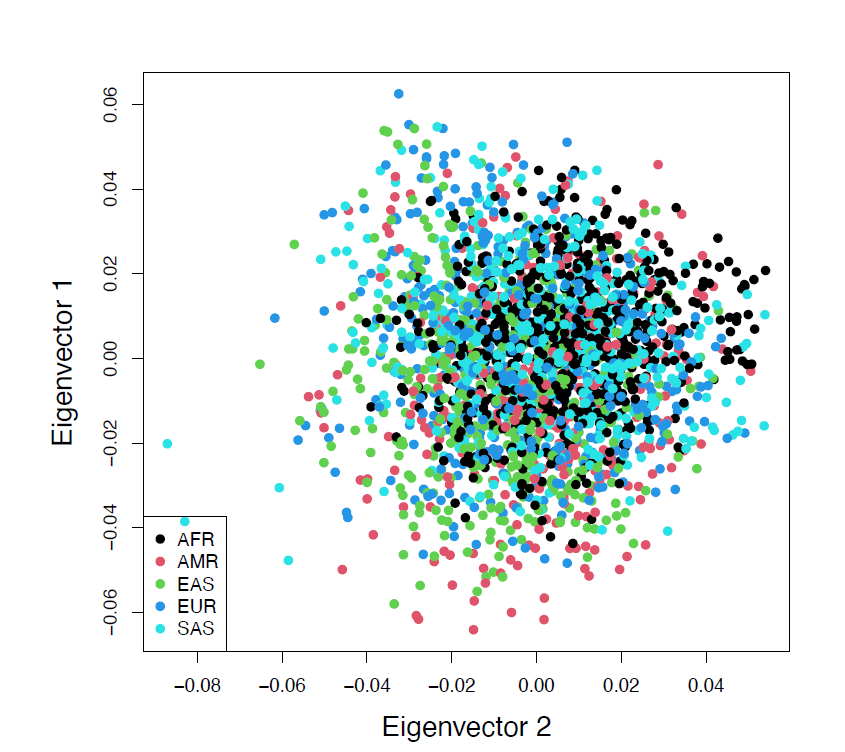


**Supplemental figure 2.** PCA plot of markers in assay (right) as well as PCA plot of 35 random selected indels in the HLA region (left). The right PCA plot show distinct population dependence, the left does not.


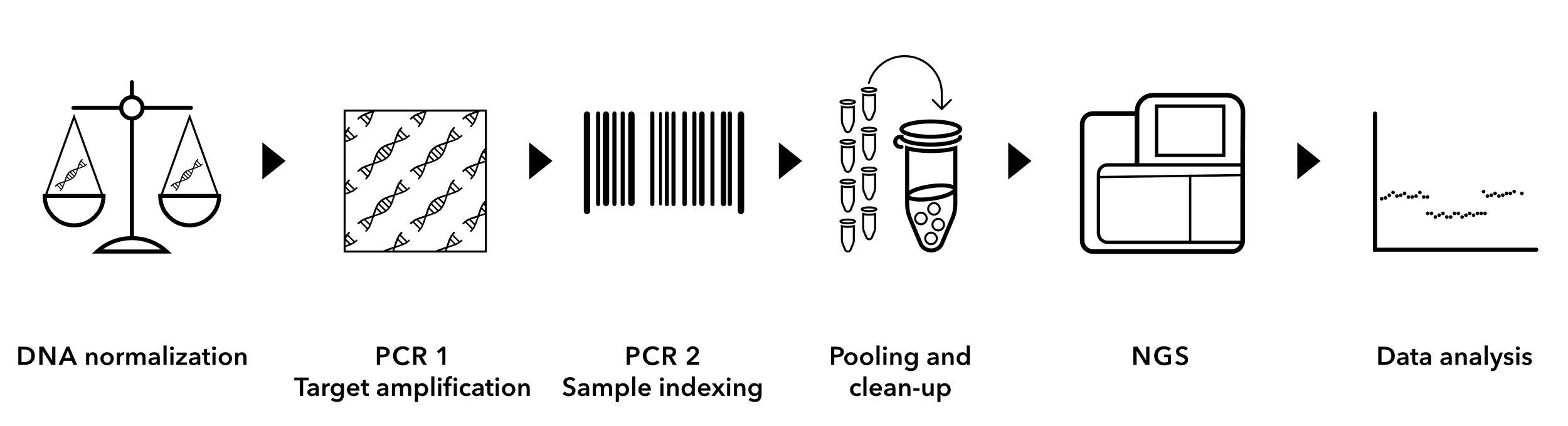


**Supplemental figure 3.** Workflow of One Lambda Devyser Chimerism and HLA loss assay
